# First Successful Isolated Intestinal Transplant in a Brazilian Series

**DOI:** 10.6061/clinics/2021/e3016

**Published:** 2021-10-20

**Authors:** Wellington Andraus, Rafael Soares Pinheiro, Allana Christina Fortunato, Flavio Henrique Ferreira Galvão, Rubens Macedo Arantes, Daniel Reis Waisberg, Andre Dong Lee, Mariana Hollanda Martins da Rocha, Lucas Souto Nacif, Vinicius Rocha Santos, Liliana Ducatti, Rodrigo Bronze de Martino, Luciana Bertocco de Paiva Haddad, Ryan Yukimatsu Tanigawa, Regis O.F. Bezerra, Alice Tung Wan Song, Luiz Augusto Carneiro-D'Albuquerque

**Affiliations:** I Disciplina de Transplante de Figado e Orgaos do Aparelho Digestivo, Departamento de Gastroenterologia, Hospital das Clinicas HCFMUSP, Faculdade de Medicina, Universidade de Sao Paulo, Sao Paulo, SP, BR.; II Unidade de Nutrologia, Departamento de Gastroenterologia, Hospital das Clinicas HCFMUSP, Faculdade de Medicina, Universidade de Sao Paulo, Sao Paulo, SP, BR.; III Disciplina de Anatomia Patologica, Hospital das Clinicas HCFMUSP, Faculdade de Medicina, Universidade de Sao Paulo, Sao Paulo, SP, BR.; IV Departamento de Radiologia, Instituto do Cancer do Estado de Sao Paulo (ICESP), Hospital das Clinicas HCFMUSP, Faculdade de Medicina, Universidade de Sao Paulo, Sao Paulo, SP, BR.

Intestinal failure (IF) is the inability to maintain nutritional and hydroelectrolytic support in the digestive tract (1-3). Patients with irreversible IF depend lifelong on total parenteral nutrition (PN).

The advent of PN has contributed to longer survival and better quality of life (QoL) for these patients; however, the prolonged use of PN can lead to complications that may result in failure of nutritional therapy, infections, and vein thrombosis. Currently, intestinal transplantation (IT) is the treatment of choice for most patients with IF who develop complications related to PN.

The most common etiology of IF is short bowel syndrome, accounting for 35%-75% of cases (4,5), and it is generally a consequence of multiple resections of different parts of the intestine.

In 1968, Okumura et al. (6) made the first attempt at IT in adults at the Hospital das Clinicas of the University of São Paulo (HC-FMUSP). From 1964 to 1970, eight other attempts were made in this direction, but the results were poor, with only one patient surviving for more than 30 days (7). The negative results of these patients were attributed to technical complications, infections, and immunosuppression (7).

In 1990, tacrolimus (FK), a more potent calcineurin inhibitor than cyclosporine, was introduced in clinical practice. This new medication led to a significant improvement in the survival rates of transplant patients and organs because of better rejection control. In 2001, IT was accepted in the USA as a therapeutic modality for patients with irreversible intestinal failure who had complications related to PN. However, the number of transplants performed remained few because of the complexity of the entire procedure. In Latin America, it was only after 2004 that a few units of IT were established in countries such as Argentina, Brazil, and Colombia (8). In Brazil, only a few attempts of isolated IT have been made, but without success.

Nevertheless, this scenario has since changed; here, we report the first successful series of isolated IT in Brazil.

The patients were operated at HC-FMUSP between 2017 and 2021. Four patients underwent isolated IT because of short bowel syndrome.

The IT technique included anastomoses of the superior mesenteric artery (SMA) and superior mesenteric vein, with the aorta and vena cava, respectively, interposed by an iliac graft from the same deceased donor. After graft revascularization, intestinal anastomoses were performed with the intestinal remnant of the recipient (duodenum, ileum, or colon). The transplanted intestine was carefully fixed, and the mesenteric gaps were closed. At the end of the surgery, a three-lumen tube was passed through the gastrostomy for gastric drainage and enteral nutrition. The triple-lumen enteral tube includes a distal jejunal lumen for enteral nutrition infusion, a proximal gastric aspiration lumen, and an assisted air vent lumen to facilitate constant or intermittent suction of gastric contents. Temporary loop ileostomy was performed in the right hypochondrium as far as possible from the surgical wound. Ileostomy closure surgery is performed 6-12 months following transplant, if there is no rejection within the preceding 3 months.

Our immunosuppression protocol comprised induction therapy with 2 mg/kg anti-thymocyte globulin (ATG) plus 1 g intravenous bolus steroid injection (methylprednisolone). Subsequent doses of ATG were administered daily and withheld if the lymphocyte count dropped below 100 cells/mm^3^. If a dose was withheld, it was administered the next day if lymphocyte count was higher than 100 cells/mm^3^. The cumulative target dose was 10 mg/kg. Steroid tapering followed the standard protocol (200, 160, 120, 80, and 40 mg). Anti-B cell antibody treatment (rituximab 150 mg/m^2^) was administered on the 3^rd^ postoperative day (POD) as a single dose. The maintenance immunosuppression regimen comprised a three-drug combination: FK, everolimus, and prednisone. FK is the mainstay therapy and is initiated immediately after IT. FK was administered via the gastric tube lumen following clamping for 1h. The initial dose was 0.15 mg/kg administered four times per day to achieve an early target range for the trough level of 20-25 ng/ml. The doses were altered according to trough levels. If the FK trough level was below the target range within 5 days, the administration route was changed in a sublingual fashion. Everolimus was started after complete abdominal wall closure, targeted to a 4-6 ng/ml trough level. Prednisone (20 mg) was introduced after methylprednisolone tapering.

Infectious disease prophylaxis included systemic antibiotics at the time of anesthesia induction and was continued for 7 days after IT Standard therapy comprised vancomycin and piperacillin-tazobactam. Concurrent antifungal coverage was employed with fluconazole for 21 days. All patients received trimethoprim-sulfamethoxazole for prophylaxis against *Pneumocystis carinii* for 1 year after IT. Albendazole and ivermectin were administered systematically for parasitic infection prophylaxis. Blood cultures were collected on the day of transplantation because of the risk of line sepsis and bacterial translocation.

Cytomegalovirus (CMV) is a viral prophylaxis target. Recipients (R) with negative CMV immunoglobulin (Ig) G antibody serostatus are considered at a higher risk to develop CMV infection. Patients were stratified according to the CMV IgG status and were considered high-risk patients when an IgG-negative recipient received a graft from an IgM-positive donor. All patients received ganciclovir 5 mg/kg twice daily for 3 weeks, starting immediately after transplantation. Prophylactic ganciclovir (5 mg/kg) was administered once daily and valganciclovir 900 mg was continued for 1 year. High-risk patients additionally received CMV Ig 150 mg/kg within 72 hours of transplantation, every alternate week for 8 weeks, and then monthly for 1 year (15 doses total). Surveillance of subclinical CMV replication included weekly serum CMV polymerase chain reaction (PCR) for 12 weeks and then monthly for 1 year.

Allograft rejection was diagnosed based on clinical parameters, particularly high ileostomy output, endoscopic mucosal aspect, and histology of biopsy specimens. Rejection surveillance was based on routine ileoscopy with collection of biopsies (five samples). The first examination was performed on day 5 after IT, then twice weekly for 2 months, once a week at the 3^rd^ month, and finally once per month in the first year. Acute rejection was stratified as mild, moderate, or severe. Mild rejection depicts localized inflammatory infiltrates concentrated around the small venules in the lamina propria. The mucosa is intact, but the crypt epithelium displays evidence of injury. Further, the rate of crypt epithelial apoptosis increases, usually with more than six apoptotic bodies per 10 crypts. Moderate rejection depicts an inflammatory infiltrate that is widely dispersed within the lamina propria. Crypt damage is distributed more diffusely, usually in areas of confluent apoptosis. Focal superficial erosions may be present in the mucosa. Severe acute rejection is distinguished by a marked degree of crypt damage, usually associated with exfoliation, histological mucosal denudation, and mucosal ulceration. Treatment for rejection must be individualized, but as a general rule, mild rejection is treated with bolus steroid therapy (1 g methylprednisolone for 3-5 days). ATG is administered in cases of steroid-resistant rejection and moderate or severe rejection. Alemtuzumab is warranted in patients with poor response to ATG treatment.

The characteristics of the IT recipients and donors and the intraoperative data are presented in Table 1. Four patients underwent transplant, three of whom are alive and well (Table 1). One patient died after 19 months of follow-up because of pulmonary infection, therefore, the first three cases had 100% 19-month survival (Figure S1).

## Case 1

A 23 year-old male, who underwent surgery for intestinal malrotation at 15 days of age, presented with mid-bowel volvulus, which led to occlusion of the SMA territory and required total enterectomy. Six months later, intestinal tract reconstruction with duodenocolic anastomosis was performed. After a failed rehabilitation attempt with PN and multiple central catheter-related infections, he underwent IT in December 2017 with a graft composed solely of the small bowel. The surgery lasted 7 hours and 27 minutes. On the 6^th^ POD, enteral diet was introduced. The patient presented with worsening renal function. The FK doses were reduced, and everolimus therapy was initiated on the 10^th^ POD. An oral diet was started on the 18^th^ POD, with good acceptance. As a side effect of everolimus therapy, the patient developed serositis and pericardial effusion, requiring surgical intervention. Everolimus has since been replaced with mycophenolate sodium. The intestinal function recovered, and the patient was discharged 60 days after IT. Currently, the patient is rehabilitated on an exclusive oral diet using double immunosuppression. He did not present with hernia or abdominal defects during follow-up.

## Case 2

A 44 year-old male, with a medical history of surgical correction of sigmoid volvulus at 2 months of age, presented with acute intestinal obstruction because of adhesions. He underwent surgical intervention; however, on the 30^th^ POD, he developed new acute intestinal obstruction, which was treated by enterectomy, total colectomy, rectal stump closure, and duodenostomy. After 1 year of in-hospital clinical rehabilitation, he underwent IT in February 2019, with an isolated bowel graft containing the small intestine, ileocecal valve, and ascending and transverse colon. The total surgery time was 7 hours and 14 minutes. Transplantation was uneventful. On the 15^th^ POD, an oral diet was started. Two months after transplant, the patient presented with steroid-resistant graft rejection and was successfully treated with 1.5 mg/kg/day ATG up to a total dose of 7 mg/kg. After 6 months, ileostomy closure surgery was performed; however, the patient developed acute intestinal obstruction and subsequently underwent laparotomy, which revealed internal herniation. The intestinal function fully recovered, and the patient was discharged on an exclusive oral diet, with triple immunosuppression and no hernias or abdominal defects. After 19 months of follow-up, the patient was hospitalized because of fever and dyspnea. Radiological investigation revealed pneumonia, and blood cultures identified multidrug-resistant *Klebsiella pneumoniae* carbapenemase-producing bacteria. He developed septic shock and died.

## Case 3

A 19 year-old male underwent segmental enterectomy in 2012 because of intestinal malrotation. In the postoperative period, he presented with mesenteric ischemia, which required resection of the entire intestinal segment nourished by the SMA and terminal jejunostomy. Four years later, he underwent intestinal tract reconstruction with jejunocolic anastomosis. He remained on home PN until 2020. In February 2020, he underwent IT with an isolated intestinal graft composed of the small intestine, ileocecal valve, ascending colon, and transverse colon. The total surgery time was 7 hours and 40 minutes. An oral diet was introduced on the 14^th^ POD with good acceptance. Ten months after transplantation, he presented with acute graft rejection and was successfully treated with ATG. Eleven months after transplantation, arterial conduit stenosis was identified and the patient underwent stent angioplasty. He remains rehabilitated on an exclusive oral diet using triple immunosuppression.

An important feature of this case is that the recipient’s CMV serology was negative (IgM^−^/IgG^−^), whereas the donor was IgG-positive. The recipient received ganciclovir in addition to intravenous immunoglobulin against CMV, according to our protocol for high-risk patients. After 13 months of follow-up, the patient has not presented with any signs or symptoms of CVM infection, including negative serum CMV PCR and negative immunohistochemistry findings on routine intestinal biopsies.

## Case 4

A 16 year-old male underwent extensive enterectomy because of intestinal torsion, remaining with 10 cm of the jejunum, transverse, and descending colon. For 5 years, he continued intestinal rehabilitation with PN and microelements. On February 2021, he underwent IT with an isolated intestinal graft composed of small intestine, ileocecal valve, ascending colon, and transverse colon. The total surgery time was 9 hours and 50 minutes. He was started an oral diet on the 8^th^ POD; however, he developed gastric distension and vomiting. On the 20^th^ POD, he presented with mild acute cellular rejection and was successfully treated with pulse steroid therapy for 3 days. On the 50^th^ POD, he developed moderate acute cellular rejection, and ATG treatment was initiated. After the second ATG dose, he was diagnosed with coronavirus disease 2019 (COVID-19) during routine nasal swab screening. We decided to proceed with ATG treatment, despite a second nasal swab positive for SARS-CoV-2 (causative virus of COVID-19) and mild respiratory symptoms. Rejection was successfully treated, and he had no complications related to COVID-19. Oral diet acceptance steadily improved, and he was discharged 3 months after the procedure. After 6 months of follow-up, he remained on an exclusive oral diet using triple immunosuppression.

Brazil has a solid foundation in organ transplants and has the largest number of recorded transplants performed in Latin America. In 2019, 14,943 transplants were performed across the country, and approximately 96% of the procedures were financed by the public Unified Health System (SUS) (9).

**Figure 1:**
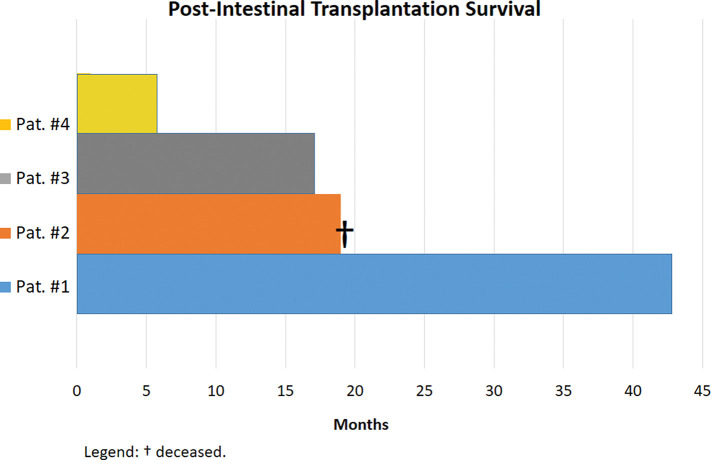
Overall survival of patients after intestinal transplantation at the Hospital das Clinicas of the University of São Paulo.

Although Brazil was a pioneer in IT with Okumura in the 60s, IT is still not a well-established modality in the country. Nonetheless, in the last 4 years, four ITs were performed in our center at HC-FMUSP with good outcomes, indicating promising prospects in the treatment of patients with IF in the future (Figure 1).

IF, although rare, is a complication that directly affects public health. It is estimated that in Brazil, approximately 800 people present with IF each year (10). These patients become dependent on daily PN and must be hospitalized, as the SUS does not finance home PN (11-13). Only a few Brazilian medical centers offer home PN through the public health system via direct agreements with the Ministry of Health; however, the available institutions are limited. Hospitalization of patients for PN results in high morbidity and mortality (14-16) and entails expenses related to hospitalization, specialized health care personnel, use of high-tech equipment, and costs of medication and secondary examinations (17). In addition, hospitalization for PN negatively impacts QoL as patients remain restricted to hospitals with limited family living along.

IT involves different modalities; although the small intestine is the main organ to be transplanted, it may be transplanted together with different organs. This type of transplant depends on the underlying disease, the quality of the other abdominal organs, the presence of liver disease, and the number of previous abdominal operations. Isolated IT is indicated in IF in the absence of severe liver dysfunction. The liver should be included in the graft in the presence of severe and irreversible liver diseases. In 2019, 81 ITs were performed in the United States, among which 55.5% required an associated liver transplant (18). When multiple abdominal organs fail, the graft must be multivisceral, and in such cases, they are transplanted en bloc: stomach, duodenum, pancreas, small intestine, and liver.

In the past decade, the overall patient and graft survival rate has improved significantly in the context of IT. Most patients have good graft function and complete restoration of oral feeding (19-21). Some centers in the USA have reached very satisfactory long-term results, with 1- and 5-year graft survival rates of 77.5% and 57.9% in the pediatric population, respectively. Among adults, the 1- and 5-year graft survival rates were 72% and 43.5%, respectively (22).

In a more recent report from the Scientific Registry of Transplant Recipients, the results from January 2014 to June 2016 showed that the 1-year graft survival varied from 60.5% to 83.0%, when including only six US centers that performed 10 or more adult IT in 2016. Including all centers that performed at least one adult IT, the mean 1-year graft survival rate in the USA was 73.6%, including all centers that performed at least one adult IT (23).

Despite the improvement in the clinical outcomes of IT, QoL should also be considered an important measure of success. The ultimate goal of any therapeutic intervention is to alleviate the disease condition and improve QoL. It is well established that QoL in IT recipients is better than that in patients with FI in PN. Rovera et al. (24) reported a significant improvement in QoL in the transplant group compared with their pre-transplant status when they were receiving PN. QoL is one of the main advantages of IT compared with PN, and, therefore, should be considered as one of the possible indications for IT.

Due to the complexity of the procedure, few centers have performed IT in the world. Usually, programs are developed in institutions where liver and other solid organ transplantation programs are already well established and have a large number of cases. Between 2005 and 2007, only 28 centers worldwide reported IT in the Intestinal Transplant Registry (25). To the best of our knowledge, this is the first successful Brazilian isolated IT series to be reported.

In conclusion, the outcomes of IT have improved in recent decades, and graft survival is now comparable to that of other solid organs. In Brazil, there has been improvement in the outcomes of IT, with the potential to benefit more patients with IF. IT is a therapeutic modality that must be offered given that intestinal rehabilitation with early restoration of nutritional autonomy has been shown to lead to better survival rate and QoL.

## Figures and Tables

**Table 1:** Characteristics of patients who underwent intestinal transplantation at Hospital das Clinicas of the University of São Paulo (HCFMUSP).

	PATIENT #1	PATIENT #2	PATIENT #3	PATIENT #4
AGE (YEARS)	25	45	27	21
SEX	MALE	MALE	MALE	MALE
WEIGHT (kg)	60	55	70	51
BMI (kg/M^2^)	19.6	18	18	19.4
CMV IgG	POSITIVE	POSITIVE	NEGATIVE	POSITIVE
BLOOD TYPE	A	O	A	O
DONOR AGE (YEARS)	14	19	30	5
DONOR SEX	MALE	MALE	FEMALE	FEMALE
DONOR WEIGHT (kg)	60	75	70	26
DONOR BMI (kg/M^2^)	20.7	25.9	24.2	20.3
DONOR CMV IgG	POSITIVE	POSITIVE	POSITIVE	POSITIVE
DONOR ICU DAYS	3	8	3	4
HLA CROSSMATCH	NEGATIVE	NEGATIVE	NEGATIVE	NEGATIVE
IF TIME (MONTHS)	24	12	96	72
ETIOLOGY	SIC	SIC	SIC	SIC
LOSS OF CENTRAL VENOUS ACCESS	4	1	0	2
TRANSPLANTATION DATE	13/12/2017	14/02/2019	05/02/2020	11/02/2021
SURGERY TIME	07:27	07:14	07:40	09:50
TOTAL ISCHEMIC TIME	07:30	06:00	5:38	07:45
COLON INCLUSION	NO	YES	YES	YES
SUPRARENAL CAVAL VEIN THROMBOSIS	YES	NO	NO	NO
ILEOSTOMY TAKE DOWN	6 M	6 M	7 M	
REJECTION	NO	YES	YES	YES
CMV INFECTION	NO	NO	NO	NO

BMI, body mass index; CMV, cytomegalovirus; ICU, intensive care unit; IF, intestinal failure; HLA, human leukocyte antigen.

**Figure S1:**
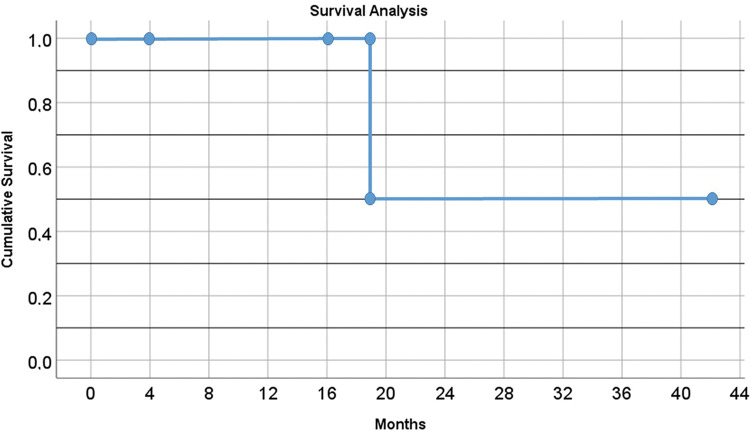
Kaplan–Meier curve.
